# Membrane Interaction Characteristics of the RTX Toxins and the Cholesterol-Dependence of Their Cytolytic/Cytotoxic Activity

**DOI:** 10.3390/ijms25063131

**Published:** 2024-03-08

**Authors:** Helena Ostolaza, Jone Amuategi

**Affiliations:** 1 Department of Biochemistry and Molecular Biology, Faculty of Science and Technology, University of the Basque Country UPV/EHU, 48080 Bilbao, Spain; jone.amuategui@ehu.eus; 2 Biofisika Institute (UPV/EHU, CSIC), 48940 Leioa, Spain

**Keywords:** RTX toxins, pore-forming toxins, lipid-protein interactions, cholesterol, cholesterol-binding motifs

## Abstract

RTX toxins are important virulence factors produced by a wide range of Gram-negative bacteria. They are secreted as water-soluble proteins that are able to bind to the host cell membrane and insert hydrophobic segments into the lipid bilayer that ultimately contribute to the formation of transmembrane pores. Ion diffusion through these pores leads then to cytotoxic and cytolytic effects on the hosts. Several reports have evidenced that the binding of several RTX toxins to the target cell membrane may take place through a high-affinity interaction with integrins of the β_2_ family that is highly expressed in immune cells of the myeloid lineage. However, at higher toxin doses, cytotoxicity by most RTX toxins has been observed also on β_2_-deficient cells in which toxin binding to the cell membrane has been proposed to occur through interaction with glycans of glycosylated lipids or proteins present in the membrane. More recently, cumulative pieces of evidence show that membrane cholesterol is essential for the mechanism of action of several RTX toxins. Here, we summarize the most important aspects of the RTX toxin interaction with the target cell membrane, including the cholesterol dependence, the recent identification in the sequences of several RTX toxins of linear motifs coined as the Cholesterol Recognition/interaction Amino acid Consensus (CRAC), and the reverse or mirror CARC motif, which is involved in the toxin–cholesterol interaction.

## 1. RTX Toxins

Pore-forming RTX toxins constitute a family of pore-forming cytotoxins/cytolysins secreted by many *Gram-negative* pathogens, including the bacteria of the genera *Actinobacillus*, *Aggregatibacter*, *Bordetella*, *Escherichia*, *Kingella*, *Mannheimia*, *Moraxella*, *Morganella*, *Pasteurella*, *Proteus*, and *Vibrio* [1,2] and are implicated in the infectious diseases caused by said pathogens. 

**Figure 1:**
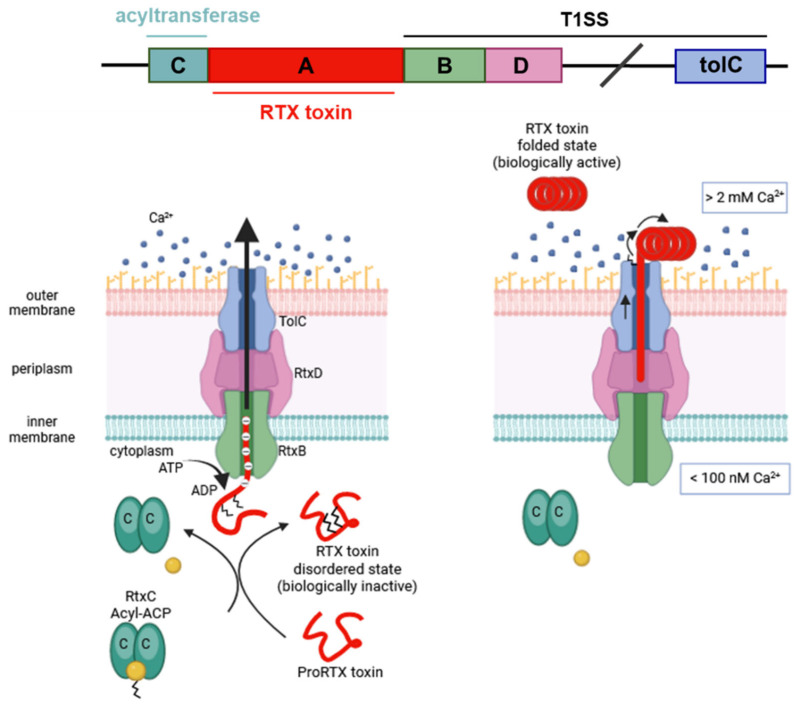
General scheme of the synthesis, post-translational modification, and secretion for the RTX toxins. The schematic organization of the operon is represented by boxes labelled from A to D. Gene product A (red) is the polypeptide corresponding to a protoxin (pro-RTX) that matures in the bacterial cytosol to the active form by post-translational acylation at two conserved internal lysine residues [5]. Fatty acylation is mediated by a specific acyltransferase encoded by the product of the gene C (dark green) and an acyl carrier protein (Acyl-ACP) [5,6,7,8,9,10]. The mature, acylated RTX toxin is then directly secreted across both membranes by the type I secretion system (T1SS) constituted by the gene products B (light green) and D (pink) and the bacterial outer membrane TolC protein [11,12,13,14,15]. Adapted from Stanley and cols. [7]. Created with Biorender.com (accessed on 10 January 2024).

RTX toxins are large protein toxins with molecular masses between ~100 and 200 kDa (*Kingella kingae* RtxA cytotoxin, 105 kDa; uropathogenic *Escherichia coli* (UPEC) α hemolysin (HlyA), 110 kDa; *Bordetella pertussis* adenylate cyclase toxin (CyaA), 177 kDa) and consist of single polypeptide chains lacking cysteine residues. Synthesis, maturation, and secretion of RTX toxins are determined by the *rtxCABD* operon [2,3,4]. Gene product A is the polypeptide corresponding to a protoxin (pro-RTX) that matures in the bacterial cytosol to the active form by post-translational acylation at two conserved internal lysine residues [5]. Fatty acylation is mediated by a specific acyltransferase encoded by the product of the gene *C* and an acyl carrier protein (Acyl-ACP) [5,6,7,8,9,10]. The mature, acylated RTX toxin is then directly secreted across both membranes by the type I secretion system (T1SS) constituted by the genes products B and D and the bacterial outer membrane TolC protein [11,12,13,14,15] (see Figure 1). There may be some minor exceptions to this general mechanism of secretion for some RTX toxins. 

**Figure 2:**
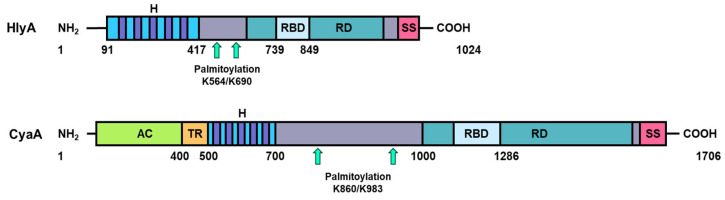
Schematic representation of the structure of the RTX toxins UPEC HlyA (top) and *B. pertussis* CyaA (bottom). These toxins consist of a pore-forming domain (H, dark blue), an acylated segment with two post-translationally acylated lysine residues (indicated with two turquois arrows), a repeat domain (RD dark green), a receptor binding domain (RBD, light blue), and a C-terminal secretion signal (SS, purple). Unlike other RTX toxins, CyaA contains a unique adenylate cyclase AC domain (AC, green) and a translocation region (TR, orange).

The mature polypeptide chain of each RTX toxin consists of four conserved regions [1,2,3,4] (Figure 2). An N-terminal hydrophobic domain harbors several putative transmembrane α-helices, which is important for insertion of the RTX toxin into the host cell membrane and pore formation [1,16,17,18,19,20,21,22,23]. This hydrophobic domain is followed by an acylation domain, a segment of variable length that contains the conserved acylated lysine residues [5,7]. The C-terminal portion of each RTX toxin contains a calcium-binding domain comprising tandem repeats of a nine-residue calcium-binding motif that upon Ca^2+^ binding adopts a “β-roll” fold [1,2,3,4,24,25,26]. The common presence of these repetitions is precisely at the origin of the historical name of the RTX protein family, where RTX stands for repeat-in-toxin [1]. The C-terminus of the toxin contains a secretion signal that is recognized by the T1SS [27,28,29]. The only exception to the arrangement of typical RTX toxins is the *B. pertussis* CyaA toxin in which an enzymatic adenylate cyclase (AC) domain is fused to the N-terminus of the RTX hemolysin via a specific linker also named translocation region (Figure 2) [2].

RTX toxins are initially expressed as inactive protoxins that require post-translational acylation to become mature toxins [2,5]. This post-translational acylation results in the addition of fatty acids with fourteen to eighteen carbons to two conserved internal lysine residues located downstream of the hydrophobic domain [5,6,7,8,9]. The fatty acids, which can be saturated, unsaturated, and hydroxylated, are attached via amide linkages to the ε-amino groups of the conserved lysines [5,9,30,31]. Fatty acylation of the RTX toxins is required for host cell cytotoxicity [7,32], but the exact role of the post-translational modification in the mechanism of action of these toxins is not fully understood. Interestingly, this activation is not necessary for the secretion of the RTX protein, since nonacylated proHlyA is secreted as efficiently as acylated HlyA [33]. The nonacylated proHlyA and proCyaA form pores in planar lipid bilayers with a much-reduced propensity, but the formed pores have apparently quite similar properties to the pores generated by the mature toxin form [34,35]. Both nonacylated proHlyA and proCyaA are also quite active in permeabilizing a naked liposome membrane [36,37], suggesting that the fatty acids are not essential for toxin penetration into the lipid bilayer. However, the post-translational modification of proRTXA is critical for the folding of the RTX toxin outside of the bacterial cell [38]. It was shown that the acyl chains covalently bound to *B. pertussis* CyaA affect toxin folding and stability [38]. Future work may demonstrate a similar role in other RTX toxins. 

In addition to the post-translational activation for exerting biological activity, all RTX toxins need to be activated by binding Ca^2+^ within the acidic glycine- and aspartate-rich nonapeptide repeats. The binding of calcium to the repeats occurs at the extracellular medium, upon toxin secretion, since toxin affinity for this cation is in the mM range, while the intracellular Ca^2+^ is about 0.1 µM [39,40,41]. It is believed that in the bacterial cytosol, the repeats domain exists in a disordered state [42]. This may allow the RTX toxin to readily pass through the T1SS machinery. Once in the extracellular medium, calcium binding to the nonapeptide repeats then appears to promote folding and adoption of a functional conformation on the secreted RTX proteins [26,43,44].

Upon release from the bacterial interior, the RTX toxins presumably interact with the target cell membrane, after which helices of the pore-forming domain insert into the lipid bilayer forming hydrophilic pores [17,19,21,45,46,47]. The uncontrolled flow of ions through these pores then leads to alterations in the target cell functions and eventually to cell lysis [2].

## 2. Interaction of the RTX Toxins with the Target Cell Membrane

Based on species and cellular specificity, RTX toxins have been historically divided into two groups, namely, the RTX hemolysins, which are capable of lysing erythrocytes and exhibit toxicity to various cell types isolated from different species, and the RTX leukotoxins, which exhibit narrow species and cell specificity because they bind via a proteinaceous receptor expressed on leukocytes [2]. Numerous reports have recently evidenced, however, that binding of almost any RTX toxin to the target cell membrane may take place through a high-affinity interaction to a proteinaceous receptor, but also in the absence of a specific protein receptor.

### 2.1. Binding through Cell-Specific Receptors

Various RTX toxins have been shown to specifically recognize several members of the β2 integrin family expressed on leukocytes [2,48,49,50,51], making the white cells highly sensitive to the cytotoxic action of those RTX toxins. This is the case of the leukotoxins LtxA and LktA secreted by *Aggregatibacter actinomycetemcomitans* and *Mannheimia haemolytica*, respectively, that use the α_L_β2 (LFA-1 or CD11a/CD18) integrin as a receptor for binding to leukocytes [50]. More recently, HlyA of UPEC *E. coli* has also been shown to bind to α_L_β2 in leukocytes [49]. The CyaA of *B. pertussis*, in turn, binds to myeloid cells through the α_M_β_2_ (Mac-1 or CD11b/CD18) integrin [48]. One of the RTX toxins of *Actinobacillus pleuropneumoniae*, ApxIIIA, also interacts with the CD18 subunit of the β_2_ integrins [51]. High-affinity binding to a proteinaceous receptor allows these toxins to be effective at very low concentrations, and it induces a variety of non-lytic effects on the target cells: disruption of bactericidal functions, stimulation or suppression of the release of pro-inflammatory cytokines, modulation of various signaling and proteolytic cascades, induction of cell cycle arrest, or activation of caspases, among others [52,53,54,55,56,57,58,59]. 

**Figure 3:**
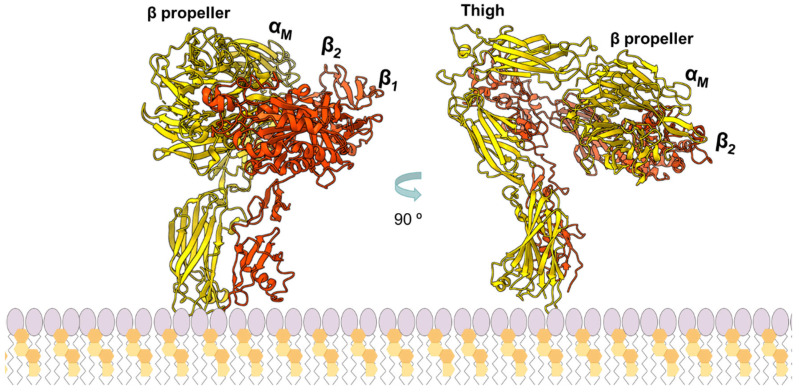
Structure of the αMβ_2_ integrin ectodomain. Structure at 2.7 Å resolution of the heterodimeric αMβ_2_ integrin published by Goldsmith and cols [62], showing the α_M_ subunit in yellow and the β_2_ subunit in red. A cartoon representation of the cell membrane lipid bilayer with phospholipid molecules (in purple) and cholesterol molecules (in orange) has been included as well. Figure redrawn from the integrin structure solved by Goldsmith and cols and deposited in PDB ID: 7USL [62].

The interaction of *B. pertussis* CyaA with α_M_β_2_ has been the most explored [60,61]. In 2001, Guermonprez and colleagues reported that CyaA uses the α_M_β_2_ integrin (CD11b/CD18) as a cell receptor in macrophages, neutrophils, and dendritic and natural killer cells [48] (Figure 3). Two years later, in 2003, the same group reported that the α_M_β_2_-binding site was localized to within residues 1166–1281 of the CyaA RTX domain [60]. More recently, and using cryoelectron microscopy, the structure at 2.7 Å resolution of a CyaA fragment (RTX751, residues 751–1706) bound to α_M_β_2_ ectodomain has been determined [62]. This structure has revealed that CyaA interacts with the headpiece (β-propeller and thigh domains) and calf-2 domain of the α_M_ subunit of the integrin in a non-canonical manner specific to bent, inactive α_M_β_2_. CyaA, in turn, engages α_M_ using the inter-blocks linkers L1 and L2 that connect, respectively, the blocks I and II, and II and III, of the calcium-binding domain. Interestingly, the binding of the RTX domain to the α_M_β_2_ ectodomain positions the two acylation sites, Lys860 and Lys983, at the plane of the host-cell membrane, with both Lys side chains pointing toward the membrane, suggesting that the essential acylations in pore-forming RTX toxins are involved in direct insertion into the target membrane. Strikingly, in this study, a non-acylated CyaA RTX fragment was used as a binding partner for the integrin. In contrast, acylation was shown to be required for a productive and tight interaction of the toxin with cells expressing CD11b [60]. Moreover, it has been reported that the presence of acyl chains in CyaA induces a significant stabilization of the apolar segments of the hydrophobic domain and of most of the acylation region so that CyaA acylation is essential for the protein refolding into its active conformation [38]. 

Regarding *A. actinomycetemcomitans* LtxA, surface plasmon resonance (SPR) experiments showed a strong affinity of LtxA for the cytosolic domains of both the CD11a and CD18 subunits, whereas the affinity of the toxin for the cytoplasmic domains of the CD11b and CD11c subunits was significantly lower [63]. More recently, the group of Dr. Welch has performed an unbiased genome-wide positive selection in a mutant library of U-937 cells [64]. The selection results have shown that the CD18 subunit is necessary and sufficient for the cytotoxic activity of HlyA, whereas all four alpha subunits are not required at all for the cytotoxic activity of the toxin. In the same study, it was shown that LtxA toxin also binds to the β_2_ integrin β subunit [64]. One interesting point is that, different to the recently discovered binding site in *B. pertussis* CyaA (linkers L1 and L2) at binding to α_M_β_2_ [62], the calcium-binding domain of the other RTX toxins do not possess inter-block linkers, raising the question as to how they bind their integrin receptors. In this regard, it was recently noted by Masin and colleagues that swapping the CyaA RTX domain for the RTX domain of *E. coli* HlyA altered the specificity of CyaA such that it required α_L_β_2_ instead of α_M_β_2_ on the target cell [65]. Thus, at least some pore-forming RTX toxins may bind to integrin receptors via RTX domains that lack linker modules.

### 2.2. Binding in the Absence of a Specific Proteinaceous Receptor

Although the high-affinity, specific interaction to β_2_ integrins converts the leukocytes as the most likely physiological targets for the RTX toxins, cytotoxicity by most RTX is also observed on β_2_-deficient cells at higher toxin doses. Moreover, for some RTX toxins such as HlyA, CyaA, and LtxA, interaction with protein-free liposomes and planar lipid bilayers has been documented [66,67,68,69]. 

Human leukocytes are 100-fold more sensitive to *E. coli* HlyA than either bladder or kidney epithelial cells [64]. However, at higher concentrations, HlyA is also cytotoxic to those renal cells, and to a wide range of hosts and cell types including erythrocytes, granulocytes, monocytes, endothelial cells, or renal epithelial cells from mice, ruminants, and primates [70,71,72,73,74,75,76]. Similarly, *A. actinomycetemcomitans* LtxA and *M. haemolytica* LktA exhibit detectable hemolytic activity on erythrocytes at high toxin concentrations [77,78], and *B. pertussis* CyaA at high concentrations can intoxicate epithelial cells, fibroblasts, and erythrocytes [2]. Another RTX toxin, the *K. kingae* RtxA toxin, is cytotoxic to synovial cells, bone osteosarcoma cells, respiratory epithelial cells, and sheep erythrocytes [79,80]. Moreover, several RTX toxins have been shown to bind and permeabilize even artificial lipid vesicles (liposomes) composed only of phospholipids [81,82,83]. All this suggests, thus, that RTX toxins may productively interact with the cell membrane through a “protein-receptor”-independent way. 

Using model lipid membranes devoid of specific proteinaceous receptors, our laboratory documented that the interaction of UPEC HlyA with the lipid bilayer appears to occur in two steps, beginning with a reversible adsorption step that is sensitive to electrostatic forces, which is followed by an irreversible membrane insertion step [84,85]. Adsorption of RTX toxins is detectable in both toxin-sensitive cells and certain toxin-resistant cells [86]. Studies with the isolated calcium-binding domain of HlyA revealed that this part of the protein adsorbs on the membrane in the early stages of HlyA–membrane interaction [87].

**Figure 4:**
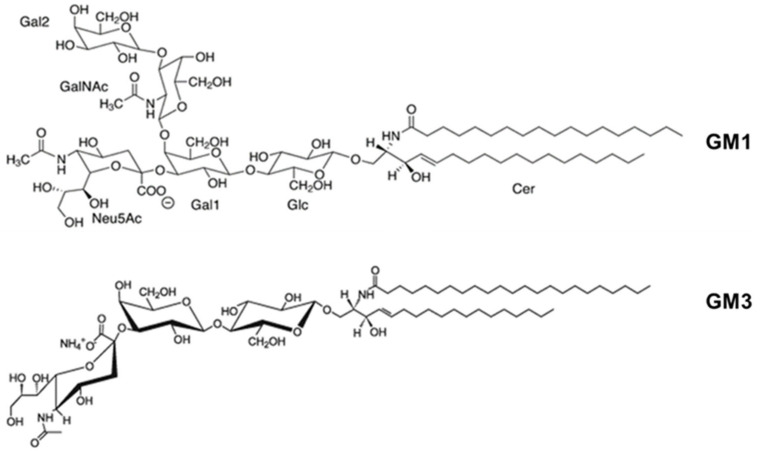
Schematic representation of the chemical structure of GM1 and GM3 gangliosides.

For several RTX toxins, it has been reported that toxin binding to the cell membrane occurs through interaction with glycans present in glycosylated lipids or proteins. In 2006, Balashova and colleagues documented that purified *A. actinomycetemcomitans* LtxA was able to lyse human and sheep erythrocytes, though the toxin concentration required to lyse erythrocytes was higher than that required to kill leukocytes, and concluded that binding to erythrocytes might be mediated by “low-affinity interaction” to some component present in the erythrocyte membrane [77]. A few years later, the same group showed that each of the five different gangliosides (GM1, GM3, GD1a, GD1b, and GT1b), containing at least one sialic acid residue, could completely block LtxA-mediated hemolysis in a dose-dependent manner [88] (Structure of GM1 and GM3 shown in Figure 4). In contrast, asialo-GM1 and free sialic acid were unable to completely block hemolysis. This suggested that the sialic acid residue is a necessary component of gangliosides required for the interaction of LtxA with erythrocytes but is not sufficient on its own to inhibit hemolysis. The results were confirmed in ganglioside-rich C6 rat glioma cells, which are recognized but not killed by LtxA and to which binding of the toxin was successfully blocked by several different gangliosides (GM1, GM3, GD1a, and GD3). In contrast, gangliosides could only partially block the LtxA-mediated killing of β_2_ integrin-expressing THP-1 cells when the ratio of gangliosides to LtxA was high and the toxin was incubated with THP-1 cells for a short incubation period [88]. The authors concluded that gangliosides act as functional receptors on erythrocytes but not on leukocytes or other cells (e.g., C6 glioma cells) [88]. By contrast, other groups reported that sialic acid residues are important for LtxA-induced cell lysis, regardless of whether the sialic acid residues are linked to the glycosylated β_2_ integrins or other glycosylated cell surface structures [89]. The authors found that preincubation of human or mouse erythrocytes with neuraminidase, an enzyme that catalyzes the hydrolysis of sialic acid residues from various substrates (glycoproteins, glycolipids, and oligosaccharides), significantly decreased LtxA-mediated hemolysis in a concentration-dependent manner. Similarly, removal of sialic acid residues significantly decreased LtxA-induced lysis of β_2_ integrin-expressing K562 cells [89]. Another group had similarly reported that β_2_ integrin-expressing Jurkat T cells pretreated with a mixture of neuraminidase and two other glycosidases, PNGase F and Endo H, were less sensitive to LtxA than untreated Jurkat T cells [90]. This suggests, hence, that in β_2_ integrin-negative cells, negatively charged sialic acid residues that are part of numerous glycosylated cell surface structures, such as glycoproteins, glycolipids, and even gangliosides may act as low-specific binding sites for LtxA. In the case of the β_2_ integrin-expressing white blood cells, it can be hypothesized that some saccharide(s) attached to the integrin molecules might be relevant in the interaction with LtxA. 

Various early reports had pointed as well to the involvement of gangliosides in the *B. pertussis* CyaA interaction with the cell membrane, even in integrin-expressing cells. Gable and colleagues (1985) showed that cytotoxicity of CyaA to polymorphonuclear leukocytes could be inhibited by pretreating the cells with neuraminidase or by preincubation of the toxin with bovine brain gangliosides [91]. Later, another laboratory showed that preincubation of CyaA with different types of gangliosides (GM1, GM3, and GT1b) inhibits the CyaA-catalyzed cAMP intoxication of CHO cells lacking CD11b/CD18 [92]. Further studies showed that the pretreatment of GM1-positive human erythrocytes and the CD11b/CD18-negative K562 cells with GM1-binding cholera toxin subunit B (CTB) decreased CyaA binding by ~30%, indicating that CTB competes with CyaA for a binding site on GM1 [93]. Another group reported that CyaA binding to several types of CD11b/CD18-expressing cells (CHO-CD11b/CD18, J774A.1, and human neutrophils) was decreased by ~80% when terminal sialic acid residues of CD11b/CD18 and other cell surface glycoproteins were removed by neuraminidase [90]. Further, they observed an almost complete loss of CyaA binding to those cells that were removed by the glycosidase PNGase F when N-linked oligosaccharides of surface glycoproteins or when N-glycosylation of newly synthesized proteins was blocked with the nucleoside antibiotic tunicamycin [90,91,92,93,94]. The authors concluded that the N-glycosylation of CR3 is crucial for the initial recognition of the integrin receptor by CyaA and subsequent cytotoxic activities of the toxin and suggested that CyaA selectively recognized sugar residues of N-linked oligosaccharides of integrins [90]. Interestingly, the ternary complex (Fab M1F5-RTX751-α_M_β_2_ ectodomain) map recently solved by cryomicroscopy also contains a well-resolved N-linked glycan at Asn1059 in α_M_ calf-2 domain, with the core fucose residue of the glycan packing onto RTX751, Leu1124, and Phe1125 residues [62]. Earlier, a study by Hasan and colleagues (2015) showed that the glutamine substitutions of asparagine residues at positions 469, 692, 801, 900, 978, 1021, and 1075 of the CD11b subunit each individually reduced binding of CyaA by about 8–44%. Further, the intracellular cAMP levels in cells expressing mutant integrins with substitutions at positions 469, 801, 978, and 1075 of CD11b were reproducibly reduced by about 30–38% after treatment with CyaA. The solved structure does not reveal, however, interactions involving any of those mutated asparagine residues, suggesting that those glycans would not be strictly required for CyaA binding to the CD11b/CD18 integrin.

For *K. kingae* RtxA toxin, Rahman and colleagues have recently reported that the toxin depends on oligosaccharides present on the host cell surface for cell binding [95]. The authors noted that pre-incubation of different cell types with neuraminidase significantly reduces the binding and cytotoxicity of RtxA. Moreover, free sialic acid partially blocked the binding of RtxA to the cells. In addition, the same authors showed that both enzymatic (PNGase F, O-glycosidase) and inhibitor-mediated (tunicamycin, benzyl-2-acetamido-2-deoxy-α-D-galactopyranoside) removal of N- or O-linked oligosaccharide chains from cell surface glycosylated structures resulted in a significant loss of RtxA binding, and deglycosylated cells were more resistant to the cytotoxic effect of RtxA than untreated cells [95]. This suggested that RtxA not only recognizes sialic acid residues but also other saccharide units of the cell surface glycoproteins on the cell surface [95]. All these results have led some authors to raise the possibility that the initial unsaturated binding of RTX cytotoxins to various cells might occur through the recognition of glycosylated membrane components, such as glycoproteins and gangliosides.

## 3. Cholesterol Dependence of the Cytolytic/Cytotoxic Activity of RTX Toxins

Cumulative pieces of evidence show that membrane cholesterol is essential for the mechanism of action of several RTX toxins. Even in the target cells that express the β2 integrins that act as specific toxin receptors, RTX toxins show cholesterol-dependence. 

For *A. actinomycetemcomitans* LtxA, removal of cholesterol from the host cell membrane with methyl-β-cyclodextrin (MβC) was shown to significantly inhibit the toxin’s ability to kill Jurkat (Jn.9) and THP-1 cells [96,97]. After replenishment of the plasma membrane cholesterol using MβC, followed by MβC-cholesterol incubation, the immune cells became again susceptible to LtxA [97]. Similarly, it was reported that a decrease in the cholesterol content of the plasma membrane of J774A.1 macrophages by MβC yielded a significant decrease in the capacity of *B. pertussis* CyaA to translocate the AC domain across the cell membrane [98]. Much earlier, in 2004, Martin and colleagues had reported that cholesterol substantially increases the rate of CyaA-induced membrane lysis, measured as the efflux of fluorescent liposomal content, in a dose-dependent manner [66]. In 2009, Herlax and colleagues showed that cholesterol-depleted erythrocytes are less sensitive to the hemolytic activity of UPEC HlyA than control erythrocytes [99]. Later, they noted that the incorporation of cholesterol into phospholipid bilayers promoted the irreversible insertion of the toxin into the membrane, which increased the toxin’s lytic activity [100]. More recently, our laboratory observed that treatment of erythrocytes with cholesterol oxidase notably reduces the CyaA-induced hemolysis [101] and that incorporation of cholesterol in pure Dipalmitoylphosphatidylcholine (DOPC) liposomes enhances the lytic capacity of CyaA on the vesicles [101]. Cholesterol is also important for the cytotoxic activity of *K. kingae* RtxA [79]. The requirement of cholesterol for toxin activity in membranes might then be another common feature shared in the family of RTX toxins.

### 3.1. Binding of the RTX Toxins to Membrane Cholesterol

Characterizing protein-cholesterol interactions is difficult due to cholesterol’s dual roles as a modulator of intrinsic protein function through direct binding (i.e., specific) and as an indirect (i.e., nonspecific) effector of membrane fluidity [102,103,104,105,106,107]. 

For a number of RTX toxins, it has been documented that the cholesterol dependence for the biological activity is sterol-specific, and it is due to direct toxin binding to the sterol molecules in the membrane and not to indirect effects of this lipid on the physical state of the phospholipid bilayer [69,79,100,108]. In 2014, Vazquez and colleagues, using different biochemical and biophysical assays, demonstrated the direct interaction of UPEC HlyA with cholesterol but not with sphingomyelin [100]. Previously, using surface plasmon resonance (SPR), Brown and colleagues determined that *A. actinomycetemcomitans* LtxA’s affinity for cholesterol-containing membranes was approximately four orders of magnitude greater than for cholesterol-free membranes [79]. *K. kingae* RtxA cytolysin was also shown to specifically bind cholesterol [69]. The authors showed that RtxA preincubated with free cholesterol exhibited significantly reduced capacity to lyse erythrocytes. They also observed a strong binding of fluorescently labeled RtxA to giant unilamellar vesicles (GUVs) composed of 75% 1-palmitoyl-2-oleoyl-*sn*-glycero-3-phosphocholine (POPC) and 25% cholesterol, whereas the binding of the toxin to GUVs composed of 100% POPC was rather weak. SPR measurements then showed a stronger affinity of RtxA for the cholesterol-containing POPC membrane than for the pure POPC membrane. Moreover, RtxA bound with 2–3-fold higher efficacy to the wells of an ELISA plate coated with cholesterol-BSA than to the wells coated with free BSA, showing that the toxin is able to interact with cholesterol independently of the presence of other membrane components [66]. Our group has recently shown that pre-incubation of *B. pertussis* CyaA with free cholesterol notably diminishes the toxin-induced hemolysis, and that binding of CyaA to pure lipid vesicles is notably increased proportionally to the cholesterol concentration present in the lipid bilayer (10–50%) and that ergosterol (ergosta-5,7,22-trien-3β-ol), an analog of cholesterol, cannot reproduce this effect [108]. 

Regarding the direct interaction of RTX toxins with cholesterol, two things should be underlined. One is that the great variability in the equilibrium dissociation constant (K_D_) values determined by several groups (mainly using SPR) for the interaction of cholesterol with different RTX toxins, which differ in many orders of magnitude, from 10^−5^ to 10^−12^ M (Summarized in Table 1). The second point is that the values of equilibrium constants (K_d_) determined for cholesterol-binding are in some cases smaller (thus, with greater affinity) than the values determined for the toxin’s association with the reported specific β_2_ integrin receptors. For *A. actinomycetemcomitans* LtxA, the maximal affinity was obtained for membranes containing 40% cholesterol using SPR with a value of K_D_ ≈ 10^−12^ M, approximately four orders of magnitude greater than the affinity determined for cholesterol-free membranes (K_D_ ≈ 10^−8^ M) [69]. Using Differential Scanning Calorimetry (DSC), the same group determined the K_D_ of association of LtxA with POPC 8.75 × 10^−4^ M, while the K_D_ for the interaction between LtxA and cholesterol was determined to be 2.31 × 10^−10^ M, which was six orders of magnitude more favorable than the affinity of LtxA for POPC. Comparatively, the K_D_ values determined by SPR for LtxA binding to the cytoplasmic domains of both α_L_ and β_2_ integrin chains were K_D_ = 15 and 4.2 × 10^−9^ M, respectively, and for the cytoplasmic domains of other integrin α_M_, α_X_, and β_3_ subunits (K_D_ = 400, 180, and 230 × 10^−9^ M, respectively), which were used as controls [109]. For *K. kingae* RtxA, the K_D_ for POPC membranes was approximately 1.5 × 10^−9^ M, while the K_D_ for cholesterol-containing membranes (POPC/CHOL 3:1, molar ratio) was 1.71 × 10^−10^ M [79]. In the case of UPEC HlyA, a K_D_ value of 1.6 × 10^−5^ M was determined for the interaction with liposomes composed of DOPC/CHOL (4:1 molar ratio) [100]. Comparatively, the same group determined a higher affinity for the HlyA-glycophorin interaction (K_D_ = 6.1 × 10^−7^ M) than for HlyA-CHOL [100]. Glycophorin acts as a high-affinity binding partner for HlyA in erythrocytes [110].

### 3.2. CRAC/CARC Motifs in the RTX Toxins as Possible Molecular Determinants of the Interaction with Membrane Cholesterol

If characterizing protein–cholesterol interaction is difficult, deciphering the exact molecular determinants of such interaction is even more complicated. As we summarize below, for several RTX toxins there is evidence indicating the involvement of cholesterol-recognizing motifs as determinants of the toxin–cholesterol interaction. 

**Figure 5:**
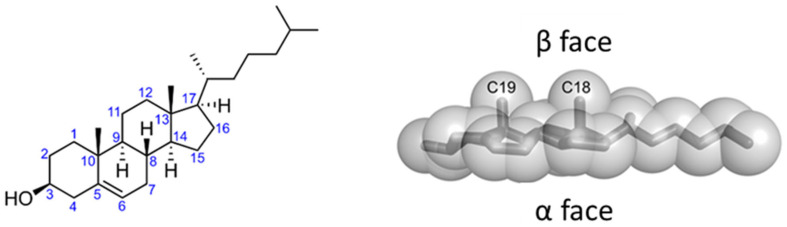
Chemical structure of cholesterol. Cholesterol has a global conical shape, and it can be divided into two parts. On the one hand, the 4-ring sterane system with the linked hydroxyl group occupies 50% of the spatial volume of the molecule, and it has very low flexibility; on the other, the terminal isooctyl chain is very flexible and, hence, it can adopt numerous conformations when bound to membrane proteins. The combination of polar (3β-hydroxyl group) and apolar (the sterol ring and the isooctyl side chain) regions impart an amphipathic nature to cholesterol, making it conducive to interaction with other membrane components (lipids and proteins). An interesting structural feature of cholesterol is the inherent asymmetry of the sterol ring plane owing to methyl substitutions on one of its faces. The smooth α face is constituted of only axial hydrogen atoms and contributes to favorable van der Waals interaction with the saturated fatty acyl chains of phospholipids. On the other hand, the rough β face characterized by the protruding methyl groups (C18 and C19) can snugly interact with the bumpy topology of a membrane protein.

Cholesterol is an amphipathic molecule derived from the sterane backbone. Its polar section is restricted to a single hydroxyl (3β_3_-OH) group, which can form two distinct types of hydrogen bonds (acceptor and donor) with a polar group belonging to either a membrane lipid or a protein. The apolar section of cholesterol, in turn, has an asymmetric structure with two distinct faces, a planar α face and a β face, which has a significantly rougher surface owing to the presence of several aliphatic groups (two methyl groups and a terminal isooctyl chain that are linked to the sterane backbone) [111] (Figure 5). Overall, these structural features open up a number of possible interactions between cholesterol and membrane lipids and proteins [112]. 

Experimental research indicates that cholesterol exhibits a more favorable interaction with sphingomyelin than with phosphatidylcholine in model lipid bilayers [113]. This preference is attributed to the presence of a saturated acyl chain in sphingomyelin compared to a cis-unsaturated chain in phosphatidylcholine [102]. The saturated chain in sphingomyelin, coupled with the trans-unsaturated sphingosine backbone, allows for maximal van der Waals interactions with cholesterol, resulting in the formation of condensed cholesterol/sphingolipids complexes [114] adopting a specific liquid-ordered (Lo) phase [111,115]. It is important to note that while cholesterol is enriched in “nanodomains” or lipid “rafts” [116,117], it is also present outside these nanodomains in the liquid-disordered (Ld) phase of the plasma membrane, containing high amounts of glycerophospholipids like phosphatidylcholine [102]. Some authors have named this pool as “accessible cholesterol” [118]. Phosphatidylcholine, unlike sphingomyelin, possesses carbonyl groups serving as hydrogen bond acceptors but lacks hydrogen bond donor groups like the amino group in sphingomyelin. Consequently, the association between cholesterol and phosphatidylcholine relies on weakly discriminative van der Waals forces and limited hydrogen bond capabilities. In cholesterol-phosphatidylcholine complexes, both α and β faces of cholesterol [119] are potentially available for interaction with a transmembrane (TM) domain. Furthermore, the −OH group of cholesterol is not buried in the complex, remaining accessible for hydrogen bond formation with a TM domain. In contrast, cholesterol forms condensed complexes with sphingolipids (either sphingomyelin or glycosphingolipids), where the −OH group is available for stabilizing hydrogen bonds with the polar head group of the sphingolipid, limiting its initial availability for interaction with a TM domain. Sphingolipids typically interact with the α face of cholesterol, leaving the β face available for the TM domain [111]. In the case of pore-forming toxins that directly bind to membrane cholesterol, the atoms of the lipid that are accessible for binding are generally restricted to the hydroxyl group, which is available only in the Ld phase. In the Lo phase, cholesterol is totally masked by the polar head groups of sphingolipids (sphingomyelins and glycosphingolipids) through a well-characterized “umbrella effect” [120,121]. This is due to the formation of a hydrogen bond network that involves the –OH group of cholesterol. Therefore, there is little chance of the –OH group of raft-associated cholesterol being targeted by an extracellular protein [106,111].

**Figure 6:**
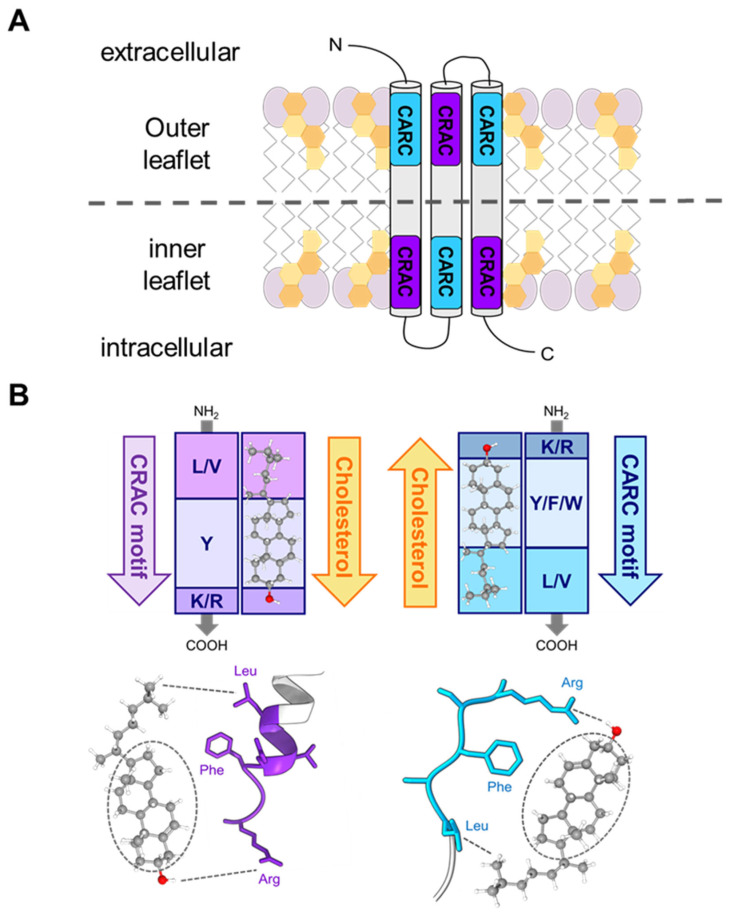
Cholesterol recognition/interaction amino acid consensus motifs. (**A**) Mirror topology of CRAC/CARC motifs within the same TM domain of a multispanning membrane-protein. The dashed line indicates the border between the inner and outer leaflets. Many cholesterol-binding proteins possess an amino acid sequence in the juxtamembrane region conforming to the pattern (L/V)-X_1–5_-(Y)-X_1–5_-(K/R) (CRAC) or the opposite (K/R)-X_1–5_-(Y/F/W)-X_1–5_-(L/V) (CARC). (**B**) Schematic illustration of the interaction between the cholesterol molecule and particular amino acid residues corresponding to CRAC/CARC-like motifs. The CRAC/cholesterol complex displays a parallel head-to-head/tail-to-tail geometry [127,128]. The branched N-terminal Leu or Val apolar residues bind to the isooctyl chain through van der Waals interactions (London forces); the mandatory aromatic residue (Tyr, Phe, or Trp) stacks onto one of the steranes four rings (CH-π stacking); and the C-terminal Lys or Arg polar residues establish hydrogen bonds with the hydroxyl group of CHOL [127]. The aromatic residues are able to stack onto the smooth α face of cholesterol or intercalate between the aliphatic spikes that emerge from the rough β face. The position of the aromatic residue is determined by the length of the X_1–5_ linkers [127,128]. Redrawn from Fantini and colleagues [127].

Different types of protein structures capable of interacting directly with membrane cholesterol have been documented, either in integral membrane proteins or in numerous bacterial toxins and viral proteins that interact with cholesterol. The presence of specific cholesterol interaction sites in bacterial toxins and viral proteins reinforces the idea that such motifs could have evolved as mechanisms for selective targeting of eukaryotic membranes. Two of the best-explored cholesterol-binding motifs are the so-called cholesterol recognition/interaction amino acid consensus CRAC and the reverse CARC motifs [107,122,123] (Figure 6). The CRAC motif is a short linear segment of 5–13 amino acids that are generally present at the end of TM helices and fulfills very simple biochemical rules to associate with cholesterol. It is defined by the following algorithm in the direction N- to C-terminus: a branched apolar leucine or valine residue, followed by a segment containing 1–5 of any residue, then an aromatic residue, tyrosine, then again a segment containing 1–5 of any residues, and finally a basic lysine or arginine (L/V)-X_1–5_-(Y)-X_1–5_-(R/K) [122]. The CARC motif, in turn, corresponds to the (R/K)-X_1–5_-(Y/F/W)-X_1–5_-(L/V) pattern [107]. Unlike CRAC, the CARC motif can accept tyrosine, phenylalanine, or tryptophan as the central amino acid residue [124], and in this case, the lysine or arginine polar residues are found in the N-terminus. Nevertheless, both motifs share a similar organization, and thus, the biochemical rules that apply to the CRAC-cholesterol interactions also apply to CARC [106,125,126,127]. 

In the last ten years, the presence of a variable number of both CRAC and CARC motifs in the primary structure has been documented for an increasing number of RTX toxins [69,79,100,108] (see Table 2). The first RTX toxin in which CRAC/CARC cholesterol-binding sites were identified was *A. actinomycetemcomitans* LtxA [66]. Primary sequence analysis revealed two CRAC motifs with the pattern (L/V)-X_1–5_-(**Y**)-X_1–5_-(R/K): one within the pore-forming domain, the CRAC^336^ (^333^LEE**Y**SKR^339^) site, which is highly conserved among RTX toxins except for *B. pertussis* CyaA, and a second one, upstream the hydrophobic domain, the CRAC^503^ (^501^VD**Y**LK^505^) site, which is unique to LtxA [69]. If the search algorithm is broadened to also include CARC sites, one CARC motif, the CARC^346^ (^340^RFKKFG**Y**NGDSL^351^) site, which is immediately posterior to the CRAC^336^ motif, can also be predicted in the LtxA pore-forming region (see Table 3). In 2014, Vazquez and colleagues identified twenty potential cholesterol-binding motifs, seven CRAC motifs, and thirteen CARC sites in the full-length sequence of UPEC HlyA; from them, four CRAC and three CARC sites are located in the pore-forming domain of HlyA [100]. In the case of *K. kingae* RtxA, five potential cholesterol-binding sites located within or adjacent to the predicted pore-forming domain (residues 140 to 410) were identified [79]. Two of them are CRAC sites, CRAC^54^ (^48^LTIPKD**Y**DIEK^58^) and CRAC^352^ (^349^LAE**Y**QR^354^), and three are CARC motifs, CARC^285^ (^280^KAISS**Y**VL^287^), CARC^343^ (^340^KFG**Y**DGDSL^348^), and CARC^448^ (^444^RHAH**Y**LERNL^453^). In 2017, Masin and colleagues predicted five CRAC motifs in the pore-forming domain of the CyaA sequence, the CRAC_627–638_ (^627^VQQSH**Y**ADQLDK^638^), CRAC_654–661_ (LAQL**Y**RDK), CRAC_721–728_ (^721^LAND**Y**ARK^728^), and CRAC_732–741_ (^732^LGGPQA**Y**FEK^741^) sites [129]. More recently, our laboratory has identified four additional CRAC/CARC sites in the *B. pertussis* CyaA primary structure: two of them in two helices of the pore-forming domain, the CRAC^521^ and CARC^532^ motifs, and the other two sites, CARC^415^ and CRAC^485^, in two helices of the translocation region [108] (see Table 3 and Table 4). 

Two lines of evidence have been used mainly to test the relationship and function of CRAC/CARC segments. One is to mutate the CRAC/CARC segment in the protein and determine if this alters function and/or interaction with cholesterol. An alternative strategy is to utilize a synthetic peptide corresponding to the CRAC/CARC site and test if the peptide has the ability to preferentially interact with cholesterol. Using full-length A. actinomycetemcomitans LtxA CRAC mutants (LtxA-CRACY336P and LtxA-CRACY503P), Brown and colleagues demonstrated in 2013 that from the two CRAC motifs identified, only the CRAC^336^ site (^333^LEE**Y**SKR^339^) located within the pore-forming domain was essential for LtxA cytotoxicity [69]. Using short peptides corresponding to both motifs they found that both bound cholesterol, but only the peptide corresponding to the CRAC site between residues 333–339 competitively inhibited the binding of LtxA to this sterol and the ability of the toxin to kill Jurkat (Jn.9) cells [69]. The authors reported K_D_ values of 2.31 × 10^−10^ M and 5.05 × 10^−8^ M for the LtxA-cholesterol and CRAC^336^ peptide-cholesterol interactions, respectively, as determined by Isothermal Titration Calorimetry (ITC) [130]. In the case of UPEC HlyA, two peptides were synthesized and analyzed, one was derived from the CARC^347^ site (RFKKLG**Y**DGDSLL, residues 341–353) located in the pore-forming domain, and the other one was from the CRAC^641^ site (VV**Y**YDK residues 639–644) from the domain between the two acylated lysines [131]. Using SPR and molecular dynamic (MD) simulations, the authors explored the interaction of both peptides with membranes of different lipid composition (POPC and POPC/CHOL at 4:1 and 2:1 molar ratios) and showed that both peptides interact preferentially with cholesterol-containing membranes, though the peptide harboring the CRAC^641^ site presented a lower K_D_ value. In addition, only this last peptide was capable of inhibiting the HlyA-induced hemolytic activity [131]. In 2018, Osickova and colleagues replaced the key tyrosine residues of the five CRAC/CARC sites identified in the hydrophobic domain of *K. kingae* RtxA, namely, Y54, Y285, Y343, Y352, and Y448, with phenylalanine residues. The authors showed that the Y54F, Y285F, and Y448F substitutions did not significantly affect the lytic activity toward erythrocytes. In contrast, the Y343F and Y352F substitutions in the CARC_340–348_ and CRAC_349–354_ motifs, respectively, reduced the lytic activity to ~50% that of wild-type RtxA [79]. Regarding B-pertussis CyaA, Masin and colleagues (2017) reported that Y/F substitutions in the respective central tyrosine residues of the four CRAC motifs (Y632, Y658, Y725, and Y738) predicted in the pore-forming domain of this toxin had no effect on the translocation or the hemolytic activity of the toxin [129]. On the contrary, our laboratory has more recently found that a single F/A substitution of the central phenylalanine residue in the four CRAC/CARC sites we identified in the first two helices of the pore-forming domain and in the translocation region very notably reduces the toxin translocation capacity and affects the hemolytic potency of CyaA [108]. In sum, it appears that the use of cholesterol recognition motifs of the CRAC/CARC type as molecular determinants for directly binding this sterol in the target cell membrane is shared by several members of the RTX toxin family. 

Using basic bioinformatics programs, it can be responded to the question of to what extent the functional CRAC/CARC motifs identified in the RTX toxins family are conserved and relevant for the action mechanisms. We have run the Clustal Omega program (EMBL-EBI, Cambridge, UK), a popular software for the alignment of multiple protein sequences, for several RTX toxins (see Supplementary Figure S1). From that exploration, it is confirmed that the CRAC_333–339_ motif firstly identified by [69] in *A. actinomycetemcomitans* LtxA is highly conserved among several RTX toxins, including HlyA, LktA, ApxIA and ApxIIA, MbxA, EhxA, MmxA, and RtxA, but not in the *B. pertussis* CyaA toxin. In addition, another two adjacently located CARC_340–348_ and CRAC_349–359_ motifs are also conserved in most of these RTX toxins, except in CyaA. The segment between residues ≈ 300–400 might thus be a kind of “hot spot” containing several conserved cholesterol-binding sites. Given that this segment is within the pore-forming domain of the respective RTX toxins, and that two hydrophobic/amphipathic α-helices can be predicted in it, which supposedly insert into the membrane, it is very tempting to speculate that cholesterol recognition through those conserved CRAC/CARC sites may be essential for proper productive insertion of those helixes into the membrane bilayer. This would explain the cholesterol dependence shown by these toxins to be cytotoxic for their target cells. Importantly, it might be a conserved mechanism shared by many RTX toxins. It is also of note that the *B pertussis* CyaA toxin seems to be a kind of *rara avis* regarding the conservation of the CRAC/CARC sites in the family. We have found that neither of the two CRAC/CARC sites (CARC_413–417_ and CRAC_481–487_) identified in the CyaA region comprising residues 400–500 (translocation region) are conserved in the rest of the RTX toxins analyzed. This may not be so strange in the end, given that this segment of the protein is involved in a function that does not exist in the rest of the RTX toxins, namely, the transport of the AC domain, and therefore could be exclusive to CyaA as believed. But more remarkable is perhaps the fact that none of the other six CRAC/CARC sites identified in the CyaA pore-forming domain [108,129] are conserved (CRAC_518–527_, CARC_527–534_, CRAC_626–636_, CRAC_653–661_, CRAC_721–728_, and CRAC_732–741_) in the rest of the RTX toxin family either (see Supplementary Figure S1). The exact meaning of the no conservation of the CRAC/CARC motifs in the CyaA sequence, and whether it may be related or not with different structural elements or different mechanisms involved in the insertion of the pore-forming domain of CyaA within the target cell membrane, is yet unknown.

## Figures and Tables

**Table 1:** Summary of the binding affinities of distinct RTX toxins to cholesterol. The RTX toxin and the interaction affinity to certain lipid compositions measured either with SPR or DSC are shown.

RTX Toxin	Lipid Composition	Affinity (M)	Measurement Technique	Reference
*A. Actinomycetemcomitans* LtxA	POPC/CHOL (3:2 molar ratio)	K_D_ = 10^−12^	SPR	Brown and colleagues [69]
	CHOL	K_D_ = 2.31 × 10^−10^	DSC	Krueger and Brown [106]
*K. kingae* RtxA	POPC/CHOL (3:1 molar ratio)	K_D_ = 1.71 × 10^−10^	SPR	Osickova and colleagues [79]
UPEC *HlyA*	DOPC/CHOL (4:1 molar ratio)	K_D_ = 1.6 × 10^−5^	SPR	Vazquez and colleagues [100]

**Table 2:** Summary of the total number of CRAC/CARC motifs identified in the primary structure of RTX toxins. Searching input algorithms used with the EMBOSS explorer FuzzPro software (https://www.bioinformatics.nl/cgi-bin/emboss/fuzzpro, (accessed on 5 March 2024), Alan Bleasby, EBI, Cambridge, UK) were as follows: [L/V]-X_1–5_-[Y/F]-X_1–5_-[K/R] for CRAC and [K/R]-X_1–5_-[Y/F]-X_1–5_-[L/V] for CARC motifs, respectively.

RTX Toxin	Bacterium	Residues	CRAC Motifs	CARC Motifs	Total No. CRAC/CARC Motifs
EhxA	Enterohemorrhagic (EHEC) *Escherichia coli*	997	16	15	31
LktA	*Mannheimia haemolytica*	953	15	13	28
PlLktA	*M. varigena*	953	21	16	37
PaxA	*Pasteurella aerogenes*	1049	19	21	40
MmxA	*Morganella morganii*	1024	13	15	28
HlyA	UPEC *E. coli*	1023	13	13	26
CyaA	*Bordetella pertussis*	1706	22	18	40
LtxA	*Aggregatibacter* *actinomycetemcomitans*	1055	24	20	44
ApxIA	*Actinobacillus* *pleuropneumoniae*	1022	18	16	34
ApxIIA	*A. pleuropneumoniae*	956	16	16	32
ApxIIIA	*A. pleuropneumoniae*	1052	22	19	41
MbxA	Moraxella bovis	927	25	18	43
RtxA	*Kingella kingae*	956	12	14	26

**Table 3:** CARC motifs identified in the sequences corresponding to the pore-forming domain of various RTX toxins. The searching input algorithm used with the EMBOSS explorer FuzzPro software (https://www.bioinformatics.nl/cgi-bin/emboss/fuzzpro, (accessed on 5 March 2024), Alan Bleasby, EBI, Cambridge, UK) was [K/R]-X_1–5_-[Y/F]-X_1–5_-[L/V]. The location of the motifs in the full-length toxin and their corresponding amino acid sequence are shown. Central phenylalanine and tyrosine residues are labeled in red.

Motif	RTX Toxin	Bacterium	Residues	Sequence
**CARC**	EhxA	EHEC *E. coli*	434–443	RHAA**F**LEDSL
			400–407	KQAM**F**EHV
			328–340	RFKKLN**Y**EGDALL
			271–278	KAVSQ**Y**IL
			102–110	KLLQK**Y**QKV
	LktA	*M. haemolytica*	405–412	KQAM**F**EHV
			333–345	RFKKLG**Y**DGDNLL
			276–287	KAVSS**Y**ILAQRV
			259–270	KVGAG**F**ELANQV
	PlLktA	*M. varigena*	405–412	KQAM**F**EHV
			333–345	RFKKLG**Y**DGDDLL
			276–287	KAVSS**Y**ILAQRV
			258–270	RKVGAG**F**ELVNQV
			221–231	KNFSG**F**SKAGL
			199–207	KINQ**F**GSKL
	PaxA	*P. aerogenes*	455–464	RHKA**F**LEDSL
			349–361	RFKKLG**Y**EGDKLL
			344–353	REFAER**F**KKL
			292–303	KAVSS**Y**ILAQRL
			275–286	KVAAG**F**ELSNQV
			115–124	RGLTL**F**APQL
	MmxA	*M. morganii*	458–469	KLLSK**Y**SEEYSV
			414–421	KQAM**F**EHV
			342–354	RFKKFG**Y**EGDSLL
			285–296	KAVSQ**Y**ILAQRV
			105–114	RGIAI**F**APQL
	HlyA	UPEC *E. coli*	457–468	KILSQ**Y**NKEYSV
			413–420	KQAM**F**EHV
			341–353	RFKKLG**Y**DGDSLL
			105–114	RGVTI**F**APQL
	CyaA	*B. pertussis*	527–534	RWAGG**F**GV
			413–420	RS**F**SLGEV
			399–410	RQDSG**Y**DSLDGV
	LtxA	*A. actinomycetemcomitans*	456–461	KL**F**NEL
			446–455	RHSA**F**LEDSL
			340–352	RFKKFG**Y**NGDSLL
			326–334	KQ**F**DRARML
			218–227	KHFGS**F**GDKL
	ApxIA	*A. pleuropneumoniae*	461–469	KE**Y**SVERVV
			410–417	KQAI**F**ERV
			338–350	RFKKFG**Y**EGDSLL
			281–292	KAVSQ**Y**IIAQRV
	ApxIIA	*A. pleuropneumoniae*	410–417	KQAM**F**EHV
			338–350	RFQKLG**Y**DGDRLL
			333–342	KSYSER**F**QKL
			281–292	KAVSS**Y**ILAQRV
	ApxIIIA	*A. pleuropneumoniae*	352–360	KLG**Y**DGDKL
			349–361	RFKKLG**Y**DGDKLL
			292–303	KAVSS**Y**ILAQRL
			275–286	KVAAG**F**ELSNQV
			115–124	RGLTL**F**APQL
	MbxA	*M. bovis*	422–431	RYAA**Y**LANNL
			417–427	KGYDSR**Y**AAYL
			387–394	KQAM**F**ESV
			317–327	RKFG**Y**DGDHLL
			258–269	KAISS**Y**VLAQRV
			240–252	KKVAAG**F**ELSNQV
			202–213	KLQNLN**F**SKTNL
	RtxA	*K. kingae*	444–453	RHAH**Y**LERNL
			409–416	KQAM**F**ESV
			339–349	KKFG**Y**DGDSLL
			280–287	KAISS**Y**VL
			262–274	KKVAAG**F**ELSNQV

**Table 4:** CRAC motifs identified in the sequences corresponding to the pore-forming domain of various RTX toxins. The searching input algorithm used with the EMBOSS explorer FuzzPro software (https://www.bioinformatics.nl/cgi-bin/emboss/fuzzpro, (accessed on 5 March 2024), Alan Bleasby, EBI, Cambridge, UK) was [L/V]-X_1–5_-[Y/F]-X_1–5_-[K/R]. The location of the motifs in the full-length toxin and their corresponding amino acid sequence are shown. Central phenylalanine and tyrosine residues are labeled in red.

Motif	RTX Toxin	Bacterium	Residues	Sequence
**CRAC**	EhxA	EHEC *E. coli*	407–414	VADK**F**AAR
			339–345	LLAA**F**HK
			322–331	LES**Y**SERFKK
			310–320	LAIADK**F**ERAK
			307–315	LS**F**LAIADK
			273–281	VSQ**Y**ILAQR
	LktA	*M. haemolytica*	401–405	LQ**Y**SK
			344–350	LLAE**Y**QR
			327–336	LES**Y**AERFKK
			312–320	LA**F**AGIADK
			278–286	VSS**Y**ILAQR
	PlLktA	*M. varigena*	401–405	LQ**Y**SK
			344–350	LLAQ**Y**QR
			327–336	LES**Y**AERFKK
			312–320	LA**F**AGIADK
			278–286	VSS**Y**ILAQR
			220–228	LKN**F**SGFSK
	PaxA	*P. aerogenes*	417–421	LE**F**SK
			353–359	LG**Y**EGDK
			331–341	LRVADN**F**NRSK
			328–332	LS**F**LR
			294–302	VSS**Y**ILAQR
			259–268	VTAS**F**TLADK
			124–133	LDK**F**LQQHSK
			117–126	LTL**F**APQLDK
	MmxA	*M. morganii*	453–462	LEDN**F**KLLSK
			324–334	LAVADK**F**KRAR
			321–329	LS**F**LAVADK
			287–295	VSQ**Y**ILAQR
	HlyA	UPEC *E. coli*	459–464	LSQ**Y**NK
			352–358	LLAA**F**HK
			323–334	LSIADK**F**KRANK
			320–328	LS**F**LSIADK
			198–210	LNNVNS**F**SQQLNK
			117–127	LLQK**Y**QKAGNK
	CyaA	*B. pertussis*	732–741	LGGPQA**Y**FEK
			721–728	LAND**Y**ARK
			653–661	LLAQL**Y**RDK
			626–638	LVQQSH**Y**ADQLDK
			518–527	VSGF**F**RGSSR
			481–487	LMTQ**F**GR
	LtxA	*A. actinomycetemcomitans*	455–464	LKL**F**NELREK
			351–357	LLGQ**F**YK
			334–343	LEE**Y**SKRFKK
			322–332	LGIAKQ**F**DRAR
			319–326	LS**F**LGIAK
			217–228	VKHFGS**F**GDKLK
			214–226	LGQVKH**F**GSFGDK
			200–209	VDT**F**SKQLNK
	ApxIA	*A. pleuropneumoniae*	455–461	LLSQ**Y**NK
			349–355	LLASF**Y**R
			332–341	LEQ**Y**SERFKK
			320–330	LNVADK**F**ERAK
			317–325	LS**F**LNVADK
			283–291	VSQ**Y**IIAQR
			247–257	VVSAS**F**ILSNK
			199–208	VDA**F**AEQLGK
			106–117	LFAPQ**F**DKLLNK
	ApxIIA	*A. pleuropneumoniae*	449–461	LQDNMK**F**LINLNK
			406–410	LE**Y**SK
			349–355	LLAD**F**HR
			342–348	LG**Y**DGDR
			331–341	LIKS**Y**SERFQK
			314–325	VTPLS**F**LNVADK
			283–291	VSS**Y**ILAQR
			195–207	VQTVDA**F**AEQISK
			101–106	LG**F**TDR
	ApxIIIA	*A. pleuropneumoniae*	417–421	LE**F**SK
			353–359	LG**Y**DGDK
			331–341	LRVADN**F**NRSK
			328–332	LA**F**LR
			294–302	VSS**Y**ILAQR
			259–268	VTAS**F**ALANK
			124–133	LDQ**F**LQKHSK
	MbxA	*M. bovis*	427–439	LANNLK**F**LSELNK
			326–332	LLAE**Y**QR
			309–318	LDE**F**AKQFRK
			294–302	LA**F**MNAADK
			203–210	LQNLN**F**SK
	RtxA	*K. kingae*	348–354	LLAE**Y**QR
			330–340	LIDE**F**AKQFKK
			316–324	LA**F**MNAADK
			224–232	LQNLPN**F**GK
			194–206	VQSIEA**F**SEQLGR

## Data Availability

Correspondence and requests for materials should be addressed to H. Ostolaza.

## Supplementary Materials

The following supporting information can be downloaded at https://www.mdpi.com/article/10.3390/ijms25063131/s1.

## References

[1] Welch R.A. (1991). Pore-Forming Cytolysins of Gram-Negative Bacteria. Mol. Microbiol..

[2] Linhartová I., Bumba L., Mašín J., Basler M., Osička R., Kamanová J., Procházková K., Adkins I., Hejnová-Holubová J., Sadílková L. (2010). RTX Proteins: A Highly Diverse Family Secreted by a Common Mechanism. FEMS Microbiol. Rev..

[3] Welch R.A. (2001). RTX Toxin Structure and Function: A Story of Numerous Anomalies and Few Analogies in Toxin Biology. Curr. Top. Microbiol. Immunol..

[4] Coote J.G. (1992). Structural and Functional Relationships among the RTX Toxin Determinants of Gram-Negative Bacteria. FEMS Microbiol. Rev..

[5] Stanley P., Packman L.C., Koronakis V., Hughes C. (1994). Fatty Acylation of Two Internal Lysine Residues Required for the Toxic Activity of *Escherichia coli* Hemolysin. Science.

[6] Balashova N.V., Shah C., Patel J.K., Megalla S., Kachlany S.C. (2009). *Aggregatibacter actinomycetemcomitans* LtxC Is Required for Leukotoxin Activity and Initial Interaction between Toxin and Host Cells. Gene.

[7] Stanley P., Koronakis V., Hughes C. (1998). Acylation of *Escherichia coli* Hemolysin: A Unique Protein Lipidation Mechanism Underlying Toxin Function. Microbiol. Mol. Biol. Rev..

[8] Barry E.M., Weiss A.A., Ehrmann I.E., Gray M.C., Hewlett E.L., Goodwin M.S. (1991). *Bordetella pertussis* Adenylate Cyclase Toxin and Hemolytic Activities Require a Second Gene, cyaC, for Activation. J. Bacteriol..

[9] Osickova A., Khaliq H., Masin J., Jurnecka D., Sukova A., Fiser R., Holubova J., Stanek O., Sebo P., Osicka R. (2020). Acyltransferase-Mediated Selection of the Length of the Fatty Acyl Chain and of the Acylation Site Governs Activation of Bacterial RTX Toxins. J. Biol. Chem..

[10] Greene N.P., Crow A., Hughes C., Koronakis V. (2015). Structure of a Bacterial Toxin-Activating Acyltransferase. Proc. Natl. Acad. Sci. USA.

[11] Holland I.B., Schmitt L., Young J. (2005). Type 1 Protein Secretion in Bacteria, the ABC-Transporter Dependent Pathway (Review). Mol. Membr. Biol..

[12] Holland I.B., Peherstorfer S., Kanonenberg K., Lenders M., Reimann S., Schmitt L. (2016). Type I Protein Secretion-Deceptively Simple yet with a Wide Range of Mechanistic Variability across the Family. EcoSal Plus.

[13] Gygi D., Nicolet J., Frey J., Cross M., Koronakis V., Hughes C. (1990). Isolation of the *Actinobacillus pleuropneumoniae* Haemolysin Gene and the Activation and Secretion of the Prohaemolysin by the HlyC, HlyB and HlyD Proteins of *Escherichia coli*. Mol. Microbiol..

[14] Wandersman C., Delepelaire P. (1990). TolC, an *Escherichia coli* Outer Membrane Protein Required for Hemolysin Secretion. Proc. Natl. Acad. Sci. USA.

[15] Glaser P., Sakamoto H., Bellalou J., Ullmann A., Danchin A. (1988). Secretion of Cyclolysin, the Calmodulin-Sensitive Adenylate Cyclase-Haemolysin Bifunctional Protein of *Bordetella pertussis*. EMBO J..

[16] Basler M., Knapp O., Masin J., Fiser R., Maier E., Benz R., Sebo P., Osicka R. (2007). Segments Crucial for Membrane Translocation and Pore-Forming Activity of Bordetella Adenylate Cyclase Toxin. J. Biol. Chem..

[17] Roderova J., Osickova A., Sukova A., Mikusova G., Fiser R., Sebo P., Osicka R., Masin J. (2019). Residues 529 to 549 Participate in Membrane Penetration and Pore-Forming Activity of the Bordetella Adenylate Cyclase Toxin. Sci. Rep..

[18] Osičková A., Osička R., Maier E., Benz R., Šebo P. (1999). An Amphipathic α-Helix Including Glutamates 509 and 516 Is Crucial for Membrane Translocation of Adenylate Cyclase Toxin and Modulates Formation and Cation Selectivity of Its Membrane Channels. J. Biol. Chem..

[19] Valeva A., Walev I., Kemmer H., Weis S., Siegel I., Boukhallouk F., Wassenaar T.M., Chavakis T., Bhakdi S. (2005). Binding of *Escherichia coli* Hemolysin and Activation of the Target Cells Is Not Receptor-Dependent. J. Biol. Chem..

[20] Benz R., Schmid A., Wagner W., Goebel W. (1989). Pore Formation by the *Escherichia coli* Hemolysin: Evidence for an Association-Dissociation Equilibrium of the Pore-Forming Aggregates. Infect. Immun..

[21] Bhakdi S., Mackman N., Nicaud J.M., Holland I.B. (1986). *Escherichia coli* Hemolysin May Damage Target Cell Membranes by Generating Transmembrane Pores. Infect. Immun..

[22] Bárcena-Uribarri I., Benz R., Winterhalter M., Zakharian E., Balashova N. (2015). Pore Forming Activity of the Potent RTX-Toxin Produced by Pediatric Pathogen *Kingella kingae*: Characterization and Comparison to Other RTX-Family Members. Biochim. Biophys. Acta.

[23] Ludwig A., Schmid A., Benz R., Goebel W. (1991). Mutations Affecting Pore Formation by Haemolysin from *Escherichia coli*. Mol. Gen. Genet..

[24] Baumann U., Wu S., Flaherty K.M., McKay D.B. (1993). Three-Dimensional Structure of the Alkaline Protease of Pseudomonas Aeruginosa: A Two-Domain Protein with a Calcium Binding Parallel Beta Roll Motif. EMBO J..

[25] Bauche C., Chenal A., Knapp O., Bodenreider C., Benz R., Chaffotte A., Ladant D. (2006). Structural and Functional Characterization of an Essential RTX Subdomain of *Bordetella pertussis* Adenylate Cyclase Toxin. J. Biol. Chem..

[26] Chenal A., Karst J.C., Sotomayor Pérez A.C., Wozniak A.K., Baron B., England P., Ladant D. (2010). Calcium-Induced Folding and Stabilization of the Intrinsically Disordered RTX Domain of the CyaA Toxin. Biophys. J..

[27] Nicaud J.-M., Mackman N., Gray L., Holland I.B. (1986). The C-Terminal, 23 kDa Peptide of *E. coli* Haemolysin 2001 Contains All the Information Necessary for Its Secretion by the Haemolysin (Hly) Export Machinery. FEBS Lett..

[28] Mackman N., Baker K., Gray L., Haigh R., Nicaud J.M., Holland I.B. (1987). Release of a Chimeric Protein into the Medium from *Escherichia coli* Using the C-Terminal Secretion Signal of Haemolysin. EMBO J..

[29] Koronakis V., Koronakis E., Hughes C. (1989). Isolation and Analysis of the C-Terminal Signal Directing Export of *Escherichia coli* Hemolysin Protein across Both Bacterial Membranes. EMBO J..

[30] Lim K.B., Walker C.R.B., Guo L., Pellett S., Shabanowitz J., Hunt D.F., Hewlett E.L., Ludwig A., Goebel W., Welch R.A. (2000). *Escherichia coli* α-Hemolysin (HlyA) Is Heterogeneously Acylated in Vivo with 14-, 15-, and 17-Carbon Fatty Acids. J. Biol. Chem..

[31] Fong K.P., Tang H.-Y., Brown A.C., Kieba I.R., Speicher D.W., Boesze-Battaglia K., Lally E.T. (2011). *Aggregatibacter actinomycetemcomitans* Leukotoxin Is Post-Translationally Modified by Addition of Either Saturated or Hydroxylated Fatty Acyl Chains. Mol. Oral Microbiol..

[32] Basar T., Havlíček V., Bezoušková S., Hackett M., Šebo P. (2001). Acylation of Lysine 983 Is Sufficient for Toxin Activity of *Bordetella pertussis* Adenylate Cyclase: Substitutions of Alanine 140 Modulate Acylation Site Selectivity of The Toxin Acyltransferase CyaC. J. Biol. Chem..

[33] Nicaud J.-M., Mackman N., Gray L., Holland I.B. (1985). Characterisation of HlyC and Mechanism of Activation and Secretion of Haemolysin from *E. coli* 2001. FEBS Lett..

[34] Masin J., Osicka R., Bumba L., Sebo P. (2015). *Bordetella* Adenylate Cyclase Toxin: A Unique Combination of a Pore-Forming Moiety with a Cell-Invading Adenylate Cyclase Enzyme. Pathog. Dis..

[35] Döbereiner A., Schmid A., Ludwig A., Goebel W., Benz R. (1996). The Effects of Calcium and Other Polyvalent Cations on Channel Formation by *Escherichia coli* Alpha-Hemolysin in Red Blood Cells and Lipid Bilayer Membranes. Eur. J. Biochem..

[36] Soloaga A., Ostolaza H., Goñi F.M., De La Cruz F. (1996). Purification of *Escherichia coli* Pro-Haemolysin, and a Comparison with the Properties of Mature α-Haemolysin. Eur. J. Biochem..

[37] Masín J., Konopásek I., Svobodová J., Sebo P. (2004). Different Structural Requirements for Adenylate Cyclase Toxin Interactions with Erythrocyte and Liposome Membranes. Biochim. Biophys. Acta.

[38] O’Brien D.P., Cannella S.E., Voegele A., Raoux-Barbot D., Davi M., Douché T., Matondo M., Brier S., Ladant D., Chenal A.A. (2019). Post-translational Acylation Controls the Folding and Functions of the CyaA RTX Toxin. FASEB J..

[39] Ostolaza H., Soloaga A., Goñi F.M. (1995). The Binding of Divalent Cations to *Escherichia coli* Alpha-Haemolysin. Eur. J. Biochem..

[40] Karst J.C., Ntsogo Enguéné V.Y., Cannella S.E., Subrini O., Hessel A., Debard S., Ladant D., Chenal A. (2014). Calcium, Acylation, and Molecular Confinement Favor Folding of *Bordetella pertussis* Adenylate Cyclase CyaA Toxin into a Monomeric and Cytotoxic Form. J. Biol. Chem..

[41] Blenner M.A., Shur O., Szilvay G.R., Cropek D.M., Banta S. (2010). Calcium-Induced Folding of a Beta Roll Motif Requires C-Terminal Entropic Stabilization. J. Mol. Biol..

[42] Chenal A., Guijarro J.I., Raynal B., Delepierre M., Ladant D. (2009). RTX Calcium Binding Motifs Are Intrinsically Disordered in the Absence of Calcium: Implication for Protein Secretion. J. Biol. Chem..

[43] Rose T., Sebo P., Bellalou J., Ladant D. (1995). Interaction of Calcium with *Bordetella pertussis* Adenylate Cyclase Toxin. Characterization of Multiple Calcium-Binding Sites and Calcium-Induced Conformational Changes. J. Biol. Chem..

[44] Rhodes C.R., Gray M.C., Watson J.M., Muratore T.L., Kim S.B., Hewlett E.L., Grisham C.M. (2001). Structural Consequences of Divalent Metal Binding by the Adenylyl Cyclase Toxin of *Bordetella pertussis*. Arch. Biochem. Biophys..

[45] Benz R. (2016). Channel Formation by RTX-Toxins of Pathogenic Bacteria: Basis of Their Biological Activity. Biochim. Biophys. Acta (BBA)-Biomembr..

[46] Hyland C., Vuillard L., Hughes C., Koronakis V. (2001). Membrane Interaction of *Escherichia coli* Hemolysin: Flotation and Insertion-Dependent Labeling by Phospholipid Vesicles. J. Bacteriol..

[47] Powthongchin B., Angsuthanasombat C. (2009). Effects on Haemolytic Activity of Single Proline Substitutions in the *Bordetella pertussis* CyaA Pore-Forming Fragment. Arch. Microbiol..

[48] Guermonprez P., Khelef N., Blouin E., Rieu P., Ricciardi-Castagnoli P., Guiso N., Ladant D., Leclerc C. (2001). The Adenylate Cyclase Toxin of *Bordetella pertussis* Binds to Target Cells via the αMβ2 Integrin (Cd11b/Cd18). J. Exp. Med..

[49] Ristow L.C., Tran V., Schwartz K.J., Pankratz L., Mehle A., Sauer J.-D., Welch R.A. (2019). The Extracellular Domain of the Β2 Integrin β Subunit (CD18) Is Sufficient for *Escherichia coli* Hemolysin and *Aggregatibacter actinomycetemcomitans* Leukotoxin Cytotoxic Activity. mBio.

[50] Lally E.T., Kieba I.R., Sato A., Green C.L., Rosenbloom J., Korostoff J., Wang J.F., Shenker B.J., Ortlepp S., Robinson M.K. (1997). RTX Toxins Recognize a Beta2 Integrin on the Surface of Human Target Cells. J. Biol. Chem..

[51] Vanden Bergh P.G.A.C., Zecchinon L.L.M., Fett T., Desmecht D. (2009). Porcine CD18 Mediates *Actinobacillus pleuropneumoniae* ApxIII Species-Specific Toxicity. Vet. Res..

[52] Confer D.L., Eaton J.W. (1982). Phagocyte Impotence Caused by an Invasive Bacterial Adenylate Cyclase. Science.

[53] Gueirard P., Le Blay K., Le Coustumier A., Chaby R., Guiso N. (1998). Variation in *Bordetella bronchiseptica* Lipopolysaccharide during Human Infection. FEMS Microbiol. Lett..

[54] Kamanova J., Kofronova O., Masin J., Genth H., Vojtova J., Linhartova I., Benada O., Just I., Sebo P. (2008). Adenylate Cyclase Toxin Subverts Phagocyte Function by RhoA Inhibition and Unproductive Ruffling. J. Immunol..

[55] Khelef N., Guiso N. (1995). Induction of Macrophage Apoptosis by *Bordetella pertussis* Adenylate Cyclase-Hemolysin. FEMS Microbiol. Lett..

[56] Pearson R.D., Symes P., Conboy M., Weiss A.A., Hewlett E.L. (1987). Inhibition of Monocyte Oxidative Responses by *Bordetella pertussis* Adenylate Cyclase Toxin. J. Immunol..

[57] Weingart C.L., Weiss A.A. (2000). *Bordetella pertussis* Virulence Factors Affect Phagocytosis by Human Neutrophils. Infect. Immun..

[58] Dhakal B.K., Mulvey M.A. (2012). The UPEC Pore-Forming Toxin α-Hemolysin Triggers Proteolysis of Host Proteins to Disrupt Cell Adhesion, Inflammatory, and Survival Pathways. Cell Host Microbe.

[59] Wiles T.J., Kulesus R.R., Mulvey M.A. (2008). Origins and Virulence Mechanisms of Uropathogenic *Escherichia coli*. Exp. Mol. Pathol..

[60] El-Azami-El-Idrissi M., Bauche C., Loucka J., Osicka R., Sebo P., Ladant D., Leclerc C. (2003). Interaction of *Bordetella pertussis* Adenylate Cyclase with CD11b/CD18: Role of Toxin Acylation and Identification of the Main Integrin Interaction Domain. J. Biol. Chem..

[61] Osicka R., Osickova A., Hasan S., Bumba L., Cerny J., Sebo P. (2014). Bordetella Adenylate Cyclase Toxin Is a Unique Ligand of the Integrin Complement Receptor 3. eLife.

[62] Goldsmith J.A., DiVenere A.M., Maynard J.A., McLellan J.S. (2022). Structural Basis for Non-Canonical Integrin Engagement by Bordetella Adenylate Cyclase Toxin. Cell Rep..

[63] Nygren P., Balashova N., Brown A.C., Kieba I., Dhingra A., Boesze-Battaglia K., Lally E.T. (2019). *Aggregatibacter actinomycetemcomitans* Leukotoxin Causes Activation of Lymphocyte Function-Associated Antigen 1. Cell. Microbiol..

[64] Ristow L.C., Welch R.A. (2019). RTX Toxins Ambush Immunity’s First Cellular Responders. Toxins.

[65] Masin J., Osickova A., Jurnecka D., Klimova N., Khaliq H., Sebo P., Osicka R. (2020). Retargeting from the CR3 to the LFA-1 Receptor Uncovers the Adenylyl Cyclase Enzyme–Translocating Segment of Bordetella Adenylate Cyclase Toxin. J. Biol. Chem..

[66] Martín C., Requero M.-A., Masin J., Konopasek I., Goñi F.M., Sebo P., Ostolaza H. (2004). Membrane Restructuring by *Bordetella pertussis* Adenylate Cyclase Toxin, a Member of the RTX Toxin Family. J. Bacteriol..

[67] Ostolaza H., Bartolomé B., Serra J.L., de la Cruz F., Goñi F.M. (1991). α-Haemolysin from *E. coli* Purification and Self-Aggregation Properties. FEBS Lett..

[68] Bakás L., Veiga M.P., Soloaga A., Ostolaza H., Goñi F.M. (1998). Calcium-Dependent Conformation of *E. coli* α-Haemolysin. Implications for the Mechanism of Membrane Insertion and Lysis. Biochim. Biophys. Acta (BBA)-Biomembr..

[69] Brown A.C., Balashova N.V., Epand R.M., Epand R.F., Bragin A., Kachlany S.C., Walters M.J., Du Y., Boesze-Battaglia K., Lally E.T. (2013). *Aggregatibacter actinomycetemcomitans* Leukotoxin Utilizes a Cholesterol Recognition/Amino Acid Consensus Site for Membrane Association. J. Biol. Chem..

[70] Keane W.F., Welch R., Gekker G., Peterson P.K. (1987). Mechanism of *Escherichia coli* Alpha-Hemolysin-Induced Injury to Isolated Renal Tubular Cells. Am. J. Pathol..

[71] Suttorp N., Flöer B., Schnittler H., Seeger W., Bhakdi S. (1990). Effects of *Escherichia coli* Hemolysin on Endothelial Cell Function. Infect. Immun..

[72] Gadeberg O.V., Orskov I. (1984). In Vitro Cytotoxic Effect of Alpha-Hemolytic *Escherichia coli* on Human Blood Granulocytes. Infect. Immun..

[73] Bhakdi S., Muhly M., Korom S., Hugo F. (1989). Release of Interleukin-1 Beta Associated with Potent Cytocidal Action of Staphylococcal Alpha-Toxin on Human Monocytes. Infect. Immun..

[74] Bhakdi S., Muhly M., Korom S., Schmidt G. (1990). Effects of *Escherichia coli* Hemolysin on Human Monocytes. Cytocidal Action and Stimulation of Interleukin 1 Release. J. Clin. Investig..

[75] Mobley H.L., Green D.M., Trifillis A.L., Johnson D.E., Chippendale G.R., Lockatell C.V., Jones B.D., Warren J.W. (1990). Pyelonephritogenic *Escherichia coli* and Killing of Cultured Human Renal Proximal Tubular Epithelial Cells: Role of Hemolysin in Some Strains. Infect. Immun..

[76] Crosby J.A., Kachlany S.C. (2007). TdeA, a TolC-like Protein Required for Toxin and Drug Export in *Aggregatibacter* (*Actinobacillus*) *actinomycetemcomitans*. Gene.

[77] Balashova N.V., Crosby J.A., Al Ghofaily L., Kachlany S.C. (2006). Leukotoxin Confers Beta-Hemolytic Activity to *Actinobacillus actinomycetemcomitans*. Infect. Immun..

[78] Murphy G.L., Whitworth L.C., Clinkenbeard K.D., Clinkenbeard P.A. (1995). Hemolytic Activity of the Pasteurella Haemolytica Leukotoxin. Infect. Immun..

[79] Osickova A., Balashova N., Masin J., Sulc M., Roderova J., Wald T., Brown A.C., Koufos E., Chang E.H., Giannakakis A. (2018). Cytotoxic Activity of *Kingella kingae* RtxA Toxin Depends on Post-Translational Acylation of Lysine Residues and Cholesterol Binding. Emerg. Microbes Infect..

[80] Kehl-Fie T.E., St. Geme J.W. (2007). Identification and Characterization of an RTX Toxin in the Emerging Pathogen *Kingella kingae*. J. Bacteriol..

[81] Ostolaza H., Bartolomé B., de Zárate I.O., de la Cruz F., Goñi F.M. (1993). Release of Lipid Vesicle Contents by the Bacterial Protein Toxin α-Haemolysin. Biochim. Biophys. Acta (BBA)-Biomembr..

[82] Brown A.C., Boesze-Battaglia K., Du Y., Stefano F.P., Kieba I.R., Epand R.F., Kakalis L., Yeagle P.L., Epand R.M., Lally E.T. (2012). *Aggregatibacter actinomycetemcomitans* Leukotoxin Cytotoxicity Occurs through Bilayer Destabilization. Cell. Microbiol..

[83] Fiser R., Konopásek I. (2009). Different Modes of Membrane Permeabilization by Two RTX Toxins: HlyA from *Escherichia coli* and CyaA from *Bordetella pertussis*. Biochim. Biophys. Acta.

[84] Bakás L., Ostolaza H., Vaz W.L., Goñi F.M. (1996). Reversible Adsorption and Nonreversible Insertion of *Escherichia coli* Alpha-Hemolysin into Lipid Bilayers. Biophys. J..

[85] Ostolaza H., Bakás L., Goñi F.M. (1997). Balance of Electrostatic and Hydrophobic Interactions in the Lysis of Model Membranes by *E. coli*α-Haemolysin. J. Membr. Biol..

[86] Iwase M., Lally E.T., Berthold P., Korchak H.M., Taichman N.S. (1990). Effects of Cations and Osmotic Protectants on Cytolytic Activity of *Actinobacillus actinomycetemcomitans* Leukotoxin. Infect. Immun..

[87] Sánchez-Magraner L., Viguera A.R., García-Pacios M., Garcillán M.P., Arrondo J.-L.R., de la Cruz F., Goñi F.M., Ostolaza H. (2007). The Calcium-Binding C-Terminal Domain of *Escherichia coli* α-Hemolysin Is a Major Determinant in the Surface-Active Properties of the Protein. J. Biol. Chem..

[88] Forman M.S., Nishikubo J.B., Han R.K., Le A., Balashova N.V., Kachlany S.C. (2010). Gangliosides Block *Aggregatibacter actinomycetemcomitans* Leukotoxin (LtxA)-Mediated Hemolysis. Toxins.

[89] Munksgaard P.S., Skals M., Reinholdt J., Poulsen K., Jensen M.R., Yang C., Leipziger J., Vorup-Jensen T., Praetorius H.A. (2014). Sialic Acid Residues Are Essential for Cell Lysis Mediated by Leukotoxin from *Aggregatibacter actinomycetemcomitans*. Infect. Immun..

[90] Morova J., Osicka R., Masin J., Sebo P. (2008). RTX Cytotoxins Recognize Beta2 Integrin Receptors through N-Linked Oligosaccharides. Proc. Natl. Acad. Sci. USA.

[91] Gable P., Eaton J., Confer D. (1985). Intoxication of Human Phagocytes by Bordetella Adenylate-Cyclase Toxin-Implication of a Ganglioside Receptor. Clin. Res..

[92] Gordon V.M., Young W.W., Lechler S.M., Gray M.C., Leppla S.H., Hewlett E.L. (1989). Adenylate Cyclase Toxins from *Bacillus anthracis* and *Bordetella pertussis*. J. Biol. Chem..

[93] Mrówczyńska L., Bobrowska-Hägerstrand M., Lindqvist C., Hägerstrand H. (2011). Bordetella Adenylate Cyclase Toxin Can Bind Ganglioside GM1. BIO.

[94] Hasan S., Osickova A., Bumba L., Novák P., Sebo P., Osicka R. (2015). Interaction of Bordetella Adenylate Cyclase Toxin with Complement Receptor 3 Involves Multivalent Glycan Binding. FEBS Lett..

[95] Rahman W.U., Osickova A., Klimova N., Lora J., Balashova N., Osicka R. (2020). Binding of *Kingella kingae* RtxA Toxin Depends on Cell Surface Oligosaccharides, but Not on Β2 Integrins. Int. J. Mol. Sci..

[96] Fong K.P., Pacheco C.M.F., Otis L.L., Baranwal S., Kieba I.R., Harrison G., Hersh E.V., Boesze-Battaglia K., Lally E.T. (2006). *Actinobacillus actinomycetemcomitans* Leukotoxin Requires Lipid Microdomains for Target Cell Cytotoxicity. Cell Microbiol..

[97] Brown A.C., Koufos E., Balashova N.V., Boesze-Battaglia K., Lally E.T. (2016). Inhibition of LtxA Toxicity by Blocking Cholesterol Binding with Peptides. Mol. Oral Microbiol..

[98] Bumba L., Masin J., Fiser R., Sebo P. (2010). Bordetella Adenylate Cyclase Toxin Mobilizes Its Beta2 Integrin Receptor into Lipid Rafts to Accomplish Translocation across Target Cell Membrane in Two Steps. PLoS Pathog..

[99] Herlax V., Maté S., Rimoldi O., Bakás L. (2009). Relevance of Fatty Acid Covalently Bound to *Escherichia coli* α-Hemolysin and Membrane Microdomains in the Oligomerization Process. J. Biol. Chem..

[100] Vazquez R.F., Maté S.M., Bakás L.S., Fernández M.M., Malchiodi E.L., Herlax V.S. (2014). Novel Evidence for the Specific Interaction between Cholesterol and α-Haemolysin of *Escherichia coli*. Biochem. J..

[101] González Bullón D., Uribe K.B., Amuategi J., Martín C., Ostolaza H. (2021). Cholesterol Stimulates the Lytic Activity of Adenylate Cyclase Toxin on Lipid Membranes by Promoting Toxin Oligomerization and Formation of Pores with a Greater Effective Size. FEBS J..

[102] Fantini J., Garmy N., Mahfoud R., Yahi N. (2002). Lipid Rafts: Structure, Function and Role in HIV, Alzheimer’s and Prion Diseases. Expert. Rev. Mol. Med..

[103] Chang H.M., Reitstetter R., Mason R.P., Gruener R. (1995). Attenuation of Channel Kinetics and Conductance by Cholesterol: An Interpretation Using Structural Stress as a Unifying Concept. J. Membr. Biol..

[104] Hanson M.A., Cherezov V., Griffith M.T., Roth C.B., Jaakola V.-P., Chien E.Y.T., Velasquez J., Kuhn P., Stevens R.C. (2008). A Specific Cholesterol Binding Site Is Established by the 2.8 A Structure of the Human Beta2-Adrenergic Receptor. Structure.

[105] Rosenhouse-Dantsker A., Noskov S., Durdagi S., Logothetis D.E., Levitan I. (2013). Identification of Novel Cholesterol-Binding Regions in Kir2 Channels. J. Biol. Chem..

[106] Fantini J., Di Scala C., Baier C.J., Barrantes F.J. (2016). Molecular Mechanisms of Protein-Cholesterol Interactions in Plasma Membranes: Functional Distinction between Topological (Tilted) and Consensus (CARC/CRAC) Domains. Chem. Phys. Lipids.

[107] Baier C.J., Fantini J., Barrantes F.J. (2011). Disclosure of Cholesterol Recognition Motifs in Transmembrane Domains of the Human Nicotinic Acetylcholine Receptor. Sci. Rep..

[108] Amuategi J., Alonso R., Ostolaza H. (2022). Four Cholesterol-Recognition Motifs in the Pore-Forming and Translocation Domains of Adenylate Cyclase Toxin Are Essential for Invasion of Eukaryotic Cells and Lysis of Erythrocytes. Int. J. Mol. Sci..

[109] Krueger E., Brown A.C. (2020). *Aggregatibacter actinomycetemcomitans* Leukotoxin: From Mechanism to Targeted Anti-Toxin Therapeutics. Mol. Oral Microbiol..

[110] Cortajarena A.L., Goñi F.M., Ostolaza H. (2001). Glycophorin as a Receptor for *Escherichia coli* α-Hemolysin in Erythrocytes. J. Biol. Chem..

[111] Fantini J., Barrantes F.J. (2009). Sphingolipid/Cholesterol Regulation of Neurotransmitter Receptor Conformation and Function. Biochim. Biophys. Acta (BBA)-Biomembr..

[112] Chaudhuri A., Chattopadhyay A. (2011). Transbilayer Organization of Membrane Cholesterol at Low Concentrations: Implications in Health and Disease. Biochim. Biophys. Acta (BBA)-Biomembr..

[113] Mattjus P., Slotte J.P. (1996). Does Cholesterol Discriminate between Sphingomyelin and Phosphatidylcholine in Mixed Monolayers Containing Both Phospholipids?. Chem. Phys. Lipids.

[114] Radhakrishnan A., Anderson T.G., McConnell H.M. (2000). Condensed Complexes, Rafts, and the Chemical Activity of Cholesterol in Membranes. Proc. Natl. Acad. Sci. USA.

[115] Brown D.A., London E. (1998). Structure and Origin of Ordered Lipid Domains in Biological Membranes. J. Membr. Biol..

[116] Simons K., Ikonen E. (1997). Functional Rafts in Cell Membranes. Nature.

[117] Anderson R.G.W., Jacobson K. (2002). A Role for Lipid Shells in Targeting Proteins to Caveolae, Rafts, and Other Lipid Domains. Science.

[118] Infante R.E., Abi-Mosleh L., Radhakrishnan A., Dale J.D., Brown M.S., Goldstein J.L. (2008). Purified NPC1 Protein. I. Binding of Cholesterol and Oxysterols to a 1278-Amino Acid Membrane Protein. J. Biol Chem.

[119] Rose I.A., Hanson K.R., Wilkinson K.D., Wimmer M.J. (1980). A Suggestion for Naming Faces of Ring Compounds. Proc. Natl. Acad. Sci. USA.

[120] Ramstedt B., Slotte J.P. (1999). Interaction of Cholesterol with Sphingomyelins and Acyl-Chain-Matched Phosphatidylcholines: A Comparative Study of the Effect of the Chain Length. Biophys. J..

[121] Fantini J., Yahi N., Garmy N. (2013). Cholesterol Accelerates the Binding of Alzheimer’s β-Amyloid Peptide to Ganglioside GM1 through a Universal Hydrogen-Bond-Dependent Sterol Tuning of Glycolipid Conformation. Front. Physiol..

[122] Li H., Papadopoulos V. (1998). Peripheral-Type Benzodiazepine Receptor Function in Cholesterol Transport. Identification of a Putative Cholesterol Recognition/Interaction Amino Acid Sequence and Consensus Pattern. Endocrinology.

[123] Jamin N., Neumann J.-M., Ostuni M.A., Vu T.K.N., Yao Z.-X., Murail S., Robert J.-C., Giatzakis C., Papadopoulos V., Lacapère J.-J. (2005). Characterization of the Cholesterol Recognition Amino Acid Consensus Sequence of the Peripheral-Type Benzodiazepine Receptor. Mol. Endocrinol..

[124] Ulmschneider M.B., Sansom M.S. (2001). Amino Acid Distributions in Integral Membrane Protein Structures. Biochim. Biophys. Acta.

[125] Fantini J., Barrantes F.J. (2013). How Cholesterol Interacts with Membrane Proteins: An Exploration of Cholesterol-Binding Sites Including CRAC, CARC, and Tilted Domains. Front. Physiol..

[126] Fantini J., Di Scala C., Evans L.S., Williamson P.T.F., Barrantes F.J. (2016). A Mirror Code for Protein-Cholesterol Interactions in the Two Leaflets of Biological Membranes. Sci. Rep..

[127] Fantini J., Epand R.M., Barrantes F.J. (2019). Cholesterol-Recognition Motifs in Membrane Proteins. Adv. Exp. Med. Biol..

[128] Azzaz F., Chahinian H., Yahi N., Di Scala C., Baier C.J., Barrantes F.J., Fantini J., Bukiya A.N., Dopico A.M. (2022). Chapter 7—Cholesterol-Recognizing Amino Acid Consensus Motifs in Transmembrane Proteins: Comparative Analysis of in Silico Studies and Structural Data. Cholesterol.

[129] Masin J., Roderova J., Osickova A., Novak P., Bumba L., Fiser R., Sebo P., Osicka R. (2017). The Conserved Tyrosine Residue 940 Plays a Key Structural Role in Membrane Interaction of Bordetella Adenylate Cyclase Toxin. Sci. Rep..

[130] Koufos E., Chang E.H., Rasti E.S., Krueger E., Brown A.C. (2016). Use of a Cholesterol Recognition Amino Acid Consensus Peptide to Inhibit Binding of a Bacterial Toxin to Cholesterol. Biochemistry.

[131] Cané L., Guzmán F., Balatti G., Daza Millone M.A., Pucci Molineris M., Maté S., Martini M.F., Herlax V. (2023). Biophysical Analysis to Assess the Interaction of CRAC and CARC Motif Peptides of Alpha Hemolysin of *Escherichia coli* with Membranes. Biochemistry.

