# Supplementary material for: Membrane Interaction Characteristics of the RTX Toxins and the Cholesterol-Dependence of Their Cytolytic/Cytotoxic Activity
Source: Int J Mol Sci. 2024 Mar 8;25(6):3131. doi: 10.3390/ijms25063131 (PMC10970614; doi:10.3390/ijms25063131)
Supplement: Supplementary file 1 [file ijms-25-03131-s001.zip › ijms-2903775-supplementary.pdf]

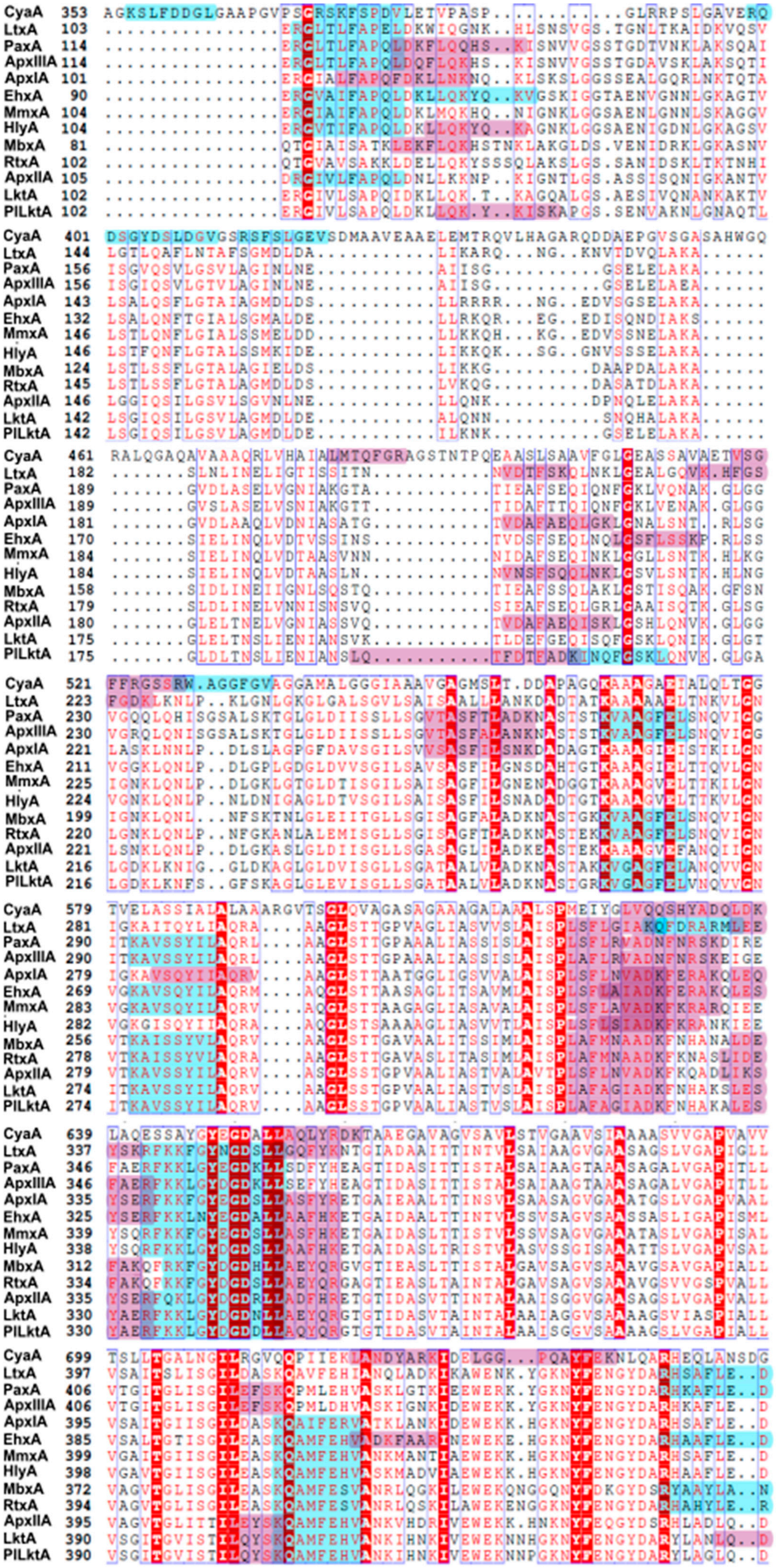
CyaA
LtxA
PaxA
ApxIIIA
ApxIA
EhxA
MmxA
HlyA
MbxA
RtxA
ApxIIA
LktA
PlLktA

Supplementary Figure S1. Alignment of the pore-forming domain of CyaA against other RTX toxins. Multiple Sequence Alignment (MSA) was performed with Clustal Omega software and plotted with ESPript 3.0. The name of the toxin and the number of the corresponding residues are shown in the left side. The strictly conserved alanine, arginine, aspartic acid, glutamic acid, glutamine, glycine, isoleucine, leucine, lysine, proline, threonine and tyrosine residues are highlighted with a red frame. CRAC motifs are depicted in purple and CARC motifs in blue.
